# Malaria incidence in Limpopo Province, South Africa, 1998–2007

**DOI:** 10.1186/1475-2875-7-162

**Published:** 2008-08-25

**Authors:** Annette AM Gerritsen, Philip Kruger, Maarten F Schim van der Loeff, Martin P Grobusch

**Affiliations:** 1 Department of Public Health, University of Venda, Private Bag x5050, Thohoyandou, 0950, Limpopo Province, South Africa; 2 Malaria Control Programme, Limpopo Department of Health and Social Development, Tzaneen, Limpopo Province, South Africa; 3 Health Service of Amsterdam, Cluster Infectious Diseases, Department of Research, PO Box 2200, 1000 CE Amsterdam, The Netherlands; 4 Infectious Diseases Unit, Division of Clinical Microbiology and Infectious Diseases, NHLS and School of Pathology, Faculty of Health Sciences, University of the Witwatersrand, 7 York Road, Parktown 2193, Johannesburg, South Africa

## Abstract

**Background:**

Malaria is endemic in the low-altitude areas of the northern and eastern parts of South Africa with seasonal transmission. The aim of this descriptive study is to give an overview of the malaria incidence and mortality in Limpopo Province for the seasons 1998–1999 to 2006–2007 and to detect trends over time and place.

**Methods:**

Routinely collected data on diagnosed malaria cases and deaths were available through the provincial malaria information system. In order to calculate incidence rates, population estimates (by sex, age and district) were obtained from Statistics South Africa. The Chi squared test for trend was used to detect temporal trends in malaria incidence over the seasons, and a trend in case fatality rate (CFR) by age group. The Chi squared test was used to calculate differences in incidence rate and CFR between both sexes and in incidence by age group.

**Results:**

In total, 58,768 cases of malaria were reported, including 628 deaths. The mean incidence rate was 124.5 per 100,000 person-years and the mean CFR 1.1% per season. There was a decreasing trend in the incidence rate over time (p < 0.001), from 173.0 in 1998–1999 to 50.9 in 2006–2007. The CFR was fairly stable over the whole period. The mean incidence rate in males was higher than in females (145.8 versus 105.6; p < 0.001); the CFR (1.1%) was similar for both sexes. The incidence rate was lowest in 0–4 year olds (78.3), it peaked at the ages of 35–39 years (172.8), and decreased with age from 40 years (to 84.4 for those ≥ 60 years). The CFR increased with increasing age (to 3.8% for those ≥ 60 years). The incidence rate varied widely between districts; it was highest in Vhembe (328.2) and lowest in Sekhukhune (5.5).

**Conclusion:**

Information from this study may serve as baseline data to determine the course and distribution of malaria in Limpopo province over time. In the study period there was a decreasing trend in the incidence rate. Furthermore, the study addresses the need for better data over a range of epidemic-prone settings.

## Background

South Africa is at the southern extreme of malaria distribution in Africa. Malaria is endemic in the low-altitude areas of the northern and eastern parts of South Africa along the border with Mozambique and Zimbabwe, with transmission taking place mainly in Limpopo, Mpumalanga and KwaZulu-Natal provinces [1]. Malaria transmission is distinctly seasonal, with transmission limited to the warm and rainy summer months (September to May), hence malaria is unstable and epidemic-prone [2].

*Plasmodium falciparum *accounts for the majority of malaria cases in southern Africa and is the predominant species associated with severe and fatal disease. Almost all South Africans lack acquired immunity, including residents of seasonal malaria transmission areas, and are, therefore, at risk for developing severe malaria [1]. In 2006, South Africa reported 12,098 cases of malaria (incidence rate 25.9 per 100,000 person-years) including 87 deaths (case fatality rate [CFR] 0.7%) [3,4]. Limpopo Province had the highest number of cases (6,369) and deaths (57) that year; the incidence rate was higher in Mpumalanga (140.1 versus 112.3 per 100,000 person-years) and the CFR was similar to that of KwaZulu-Natal (both 0.9%).

The South African Malaria Control Programme is active since 1945 in all three provinces and currently includes: (i) vector control through intensive indoor residual house-spraying (IRS); (ii) case management (diagnosis with *P. falciparum*-specific HRP-2 rapid antigen detection tests [RDTs] [5]; (iii) treatment of uncomplicated malaria with artemisinin combination therapy [ACT] in the form of artemether-lumefantrine) [6,7]; (iv) disease surveillance; (v) epidemic preparedness and response; and (vi) health promotion. The key intervention has been IRS since 1945, using both dichlorodiphenyltrichloroethane (DDT) and pyrethroids. The use of DDT has been scaled up since 2000, in order to combat insecticide-resistant *Anopheles funestus *[8].

Data are available from the computerized malaria information system from Limpopo Department of Health and Social Development from 1998–2007. These have been used for national health statistics, including reported numbers of malaria cases and incidence, and reported numbers of deaths from malaria and CFRs [3,4]. These national health statistics data are available per year and/or malaria season for Limpopo Province as a whole. No data are available for the different sexes and age groups, nor for the district council level.

Several studies have been conducted on malaria epidemiology in KwaZulu-Natal, using data from the provincial malaria information system [9-13]. The aim of this article is to give a detailed overview of malaria incidence and mortality in Limpopo Province, South Africa, for the seasons 1998–1999 to 2006–2007, based on the routinely collected provincial data. It focuses on the reported malaria cases (numbers and incidence rates) and deaths (numbers and CFRs) overall, and by sex, age group and district council.

This information is of importance as there is a basic lack of high-quality epidemiological data (for morbidity and mortality) for epidemic-prone areas [14]. In Africa, the areas with seasonal transmission (and hence with a higher risk of epidemics) are located across the Sahelean belt, down trough the horn of Africa, into East Africa and throughout Southern Africa [15].

## Methods

For this descriptive study data from the malaria information system of Limpopo Province were used. This system has been developed by the Malaria Research Programme of the South African Medical Research Council using Microsoft Access for data entry and validation [16].

Malaria is a notifiable disease in South Africa. The case reporting system aims to capture every parasitologically confirmed infection through both passive and active surveillance, although the latter has been greatly scaled down during the past decade [17]. Active surveillance consisted of screening measures by which teams went into a community with a known risk of malaria, or where there was a suspicion of parasite carriers. Smears were taken from all community members, but with emphasis on those with fever, a history of fever, a travel history to a malaria risk area, or possible migrants from malaria endemic areas. There was less need for active surveillance after the introduction of RDTs in 1998, as all suspected malaria patients could now get a blood test at the primary health care (PHC) facilities. Furthermore, the yield of active surveillance became extremely low; only 0.2% of active smears were found to be positive. The passive surveillance system did not change during the study period.

Only cases positively confirmed by either microscopy or RDT are notified and entered into the system. RDTs are available at all primary health care (PHC) facilities throughout the province, and microscopy is available in laboratories e.g. at the hospitals. All PHC facilities and hospitals (including private hospitals) notify all parasitologically confirmed infections and in the areas most at risk, the Malaria Control Programme is actively involved in this process e.g. by visiting facilities twice weekly for the collection and verification of records, or by the placement of a staff member who is responsible for the notification, and by performing regular checks. Since 1996, efforts are also made to stimulate private general practitioners to report cases: however, currently not all of them notify infections (although some of the referral laboratories do so) possibly resulting in some underreporting of malaria cases which we estimate to be less than 5% in any case. Normally it takes between 7–14 days between date of diagnosis and date of entry into the system.

**Figure 1:**
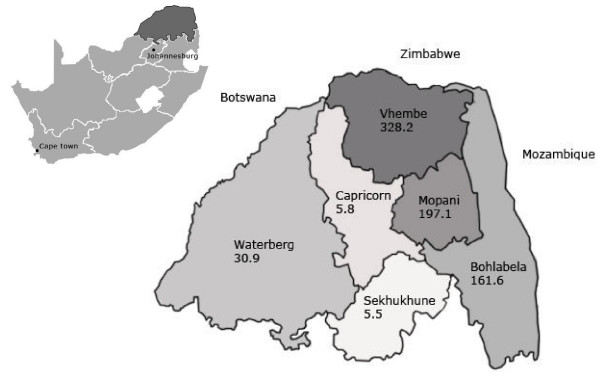
Limpopo Province districts, including mean malaria incidence rates, 1998 – 1999 to 2004 – 2005 seasons.

As malaria transmission in South Africa is seasonal, it is best presented using seasonal data. A malaria season was taken to be the period from 1 July to 30 June the following year. For the seasons 1998 – 1999 through 2006 – 2007 the following data were extracted for all reported confirmed malaria cases: date of diagnosis, surveillance type (active or passive), sex, age (in years), district council where the case resided, country or province in South Africa where the case presumably contracted malaria, and death due to malaria (yes/no). Age was grouped into five-year age categories; cases 60 years and older were aggregated into one age group. Until December 2005 Limpopo Province included six districts: Bohlabela, Capricorn, Greater Sekhukhune, Mopani, Vhembe and Waterberg (Figure 1). After that, Bohlabela district has been divided between Limpopo and Mpumalanga provinces and thus no longer exists as and administrative entity [18]. Therefore, the data analyses for the separate districts have been limited to the seasons 1998 – 1999 to 2004 – 2005. For the years 2001 to 2007 mid-year population estimates for the whole province by sex and age group were obtained from Statistics South Africa [19]. The annual population growth rates over these years were calculated. These turned out to be 1.007% per year for the total population of the province, 1.006% per year for the females and 1.008% for the males, and ranged from 0.978% to 1.041% per year for the different age groups. These percentages were then used to calculate mid-year population estimates backward in time for the years 1998, 1999 and 2000.

In order to calculate the malaria incidence rates per 100,000 person-years for the separate seasons for the province as a whole, the number of reported malaria cases per season was divided by the mean of the preceding and the following mid-year population estimates (since this corresponds to the respective mid-malaria season population) and then multiplied by 100,000. The mean incidence rate per season over the whole period of nine seasons for the province as a whole, both sexes and the different age groups was calculated as follows: first, for the specific group the total number of reported malaria cases for this period was divided by the mean of the mid-year estimates of 1998 and 2007 (as these corresponded to the population totals at the beginning and end of the period respectively). Then this number was divided by 9 (seasons), and finally multiplied by 100,000.

Population counts for the district councils were obtained from South Africa's most recent national census, which was carried out in October 2001 [20]. As this date corresponds reasonably to the middle of the period ranging from 1 July 1998 to 31 June 2005 (which actually is 31 December 2001), these population estimates were used to calculate the mean incidence rate per season over the whole period of seven seasons for the different districts.

The CFR is defined as the number of deaths due to malaria divided by the number of malaria cases and expressed as a percentage.

The Chi squared test for trend was used to test whether the malaria incidence rate was statistically significant decreasing over the malaria seasons and the CFR increasing by age group. Furthermore, the Chi squared test was used to calculate whether the differences in incidence rate and CFR between both sexes were statistically significant and whether the malaria incidence rate was statistically significant associated with age. All data analyses were conducted in SPSS (version 14.0; SPSS Inc, Chicago Ill).

## Results

In total, 58,768 cases of malaria were reported, including 628 deaths, in the seasons 1998 – 1999 to 2006 – 2007. 3,047 (5.2%) of the cases were detected by active surveillance and 55,717 (94.8%) by passive surveillance (for 4 cases no data on surveillance type were available). In Table 1, the number of reported malaria cases, the incidence rate per 100,000 person-years (including 95% confidence intervals (CI)), the number of reported malaria deaths and the CFR are given for each of the nine seasons. There were a mean 6,530 cases (standard deviation (SD) 2,236.5) and 70 deaths (SD 27.5) per season, with a mean incidence rate of 124.5 (SD 44.5) and a mean CFR of 1.1% (SD 0.3).

**Table 1:** Incidence of malaria cases and deaths, Limpopo Province, 1998 – 1999 to 2006 – 2007 seasons

Malaria season	No. of reported malaria cases	Total population	Incidence rate (per 100,000 person-years) (95% CI)	No. of reported malaria deaths	CFR (%)
1998–1999	8,833	5,106,140	173.0 (169.4 – 176.6)	105	1.2
1999–2000	8,477	5,140,225	164.9 (161.4 – 168.4)	75	0.9
2000–2001	9,942	5,174,537	192.1 (188.4 – 195.9)	85	0.9
2001–2002	6,140	5,210,191	117.8 (114.9 – 120.8)	53	0.9
2002–2003	5,132	5,246,690	97.8 (95.1 – 100.5)	62	1.2
2003–2004	6,384	5,282,444	120.9 (117.9 – 123.8)	115	1.8
2004–2005	4,893	5,318,050	92.0 (89.4 – 94.6)	48	1.0
2005–2006	6,229	5,350,748	116.4 (113.5 – 119.3)	52	0.8
2006–2007	2,738	5,384,363	50.9 (48.9 – 52.8)	33	1.2

**Figure 2:**
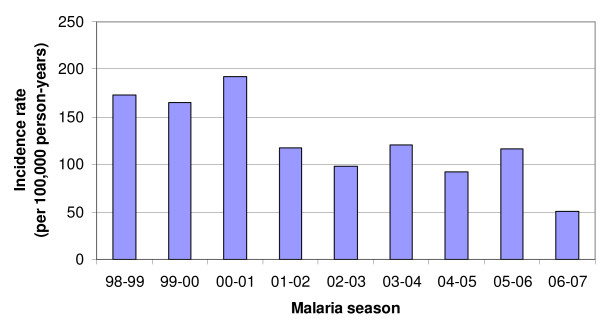
Malaria incidence rates per season, Limpopo Province, 1998 – 1999 to 2006 – 2007 seasons.

In Figure 2, the malaria incidence rates per season are graphically presented. The test for trend was highly significant (X^2 ^= 4,859.2; p < 0.001) indicating that the incidence rate of malaria is decreasing over the seasons.

Malaria transmission in the province mainly occurs from September to May, numbers of cases are very low in June, July and August. The distribution of reported malaria cases over the different months varies somewhat between the different seasons. In general, the number of cases peaked between October and April (most often in either November or January), although in some seasons there were two peaks (one smaller and one bigger).

Out of the total of 58,768 reported cases of malaria, 32,314 were among males (55%) and 26,449 among females (45%) (for five cases no data on sex were available). There were a mean 3,590 cases among males (SD 1,168.7) and 2,939 cases among females (SD 1,074.1) per season. The mean incidence rate per season was 145.8 per 100,000 person-years for males (95% CI 141.0 – 150.6) and 105.6 for females (95% CI 101.8 – 109.4), which yields a statistically significant difference (X^2 ^= 170.9; p < 0.001). Of all 628 deaths due to malaria, 336 were among males and 292 among females, with a mean of 37 deaths among males (SD 13.5) and 32 deaths among females (SD 14.8) per season. The mean CFR was similar for both males and females: 1.1% (X^2 ^= 0.567; p = 0.451).

The median age of all cases was 21 years (IQR 9.5 to 32.5 years; range 0 to 99 years) (for 310 cases data on age were missing). Table 2 gives the mean number of reported malaria cases and deaths, the mean incidence rate (95% CI) and CFR for each age category. The CFR increased statistically significantly with increasing age (test for trend, X^2 ^= 444.9; p < 0.001).

**Table 2:** Mean incidence of malaria per age category, Limpopo Province, 1998 – 1999 to 2006 – 2007 seasons

Age category (years)	Mean no. of reported malaria cases per season	Mean incidence rate (per 100,000 person-years) (95% CI)	Mean no. of reported malaria deaths per season	Mean CFR (%)
0 – 4	524	78.3 (71.6 – 85.0)	2.6	0.5
5 – 9	713	99.4 (92.2 – 106.7)	1.3	0.2
10 – 14	834	116.7 (108.8 – 124.6)	2.6	0.3
15 – 19	873	133.2 (124.4 – 142.0)	4.0	0.5
20 – 24	758	150.8 (139.5 – 160.9)	4.9	0.6
25 – 29	588	148.2 (136.2 – 160.2)	6.6	1.1
30 – 34	513	171.5 (156.7 – 186.3)	7.7	1.5
35 – 39	424	172.8 (156.3 – 189.2)	5.9	1.4
40 – 44	337	160.3 (143.2 – 177.4)	5.3	1.6
45 – 49	261	141.6 (124.5 – 158.8)	7.9	3.0
50 – 54	212	136.7 (118.3 – 155.1)	4.8	2.2
55 – 59	137	111.0 (92.4 – 129.6)	3.9	2.8
≥ 60	320	84.4 (75.1 – 93.6)	12.1	3.8

**Figure 3:**
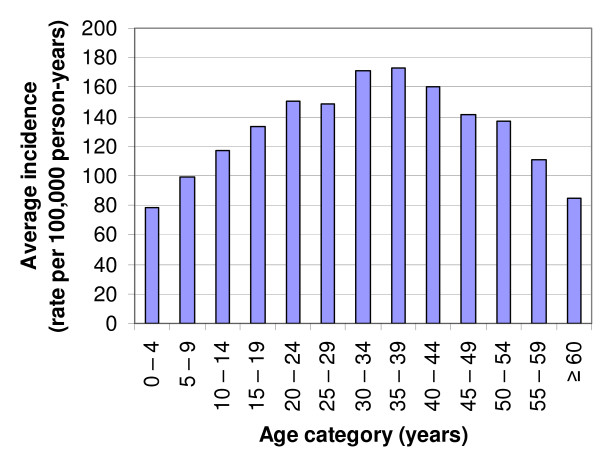
Mean malaria incidence rates per age category, Limpopo Province, 1998 – 1999 to 2006 – 2007 seasons.

In Figure 3, the mean malaria incidence rates per age category are graphically presented. The Chi squared test was highly significant (X^2 ^= 383.5; p < 0.001) indicating that there is an association between the incidence rate of malaria and age.

Table 3 gives the mean number of reported malaria cases and deaths, the mean incidence rate (95% CI) and CFR for each district council. In Figure 1 the mean incidence rates for each of the six districts are given.

**Table 3:** Mean incidence of malaria per district, Limpopo Province, 1998 – 1999 to 2006 – 2005 seasons

District council	Mean no. of reported malaria cases per season	Mean incidence rate (per 100,000 person-years) (95% CI)	Mean no. of reported malaria deaths per season	Mean CFR (%)
Bohlabela	966	161.6 (151.4 – 171.8)	11.6	1.2
Capricorn	67	5.8 (4.4 – 7.2)	2.3	3.4
Mopani	1901	197.1 (188.3 – 206.0)	19.1	1.0
Sekhukune	53	5.5 (4.0 – 7.0)	1.0	1.9
Vhembe	3938	328.2 (318.0 – 338.4)	42.2	1.1
Waterberg	190	30.9 (26.5 – 35.3)	1.1	0.6

The majority of all malaria cases reported over the whole period (37,487; 63.8%) were reported to have contracted malaria in Limpopo Province itself, 3.0% contracted malaria in Mozambique, 1.6% in Zimbabwe and 0.5% in either Mpumalanga, KwaZulu-Natal, other African countries or India. However, for many cases data on the location were malaria presumably is contracted were missing (18,297; 31.1%).

When the cases definitely originating from Limpopo are compared to "the rest" (those not originating from the province and those with an unknown location of contracting malaria), it is found that in the latter group there are significantly more males (58.8%) than in the first group (52.8%) (X^2 ^= 197.3; p < 0.001) and that the median age in the latter group is higher (24.0; IQR 13.5 – 35.5) compared to the first group (20.0; IQR 9.5 – 31.5) (Mann-Whitney U test: p < 0.001). Furthermore, while 65.6% of the first group is diagnosed in Vhembe district and 22.2% in Mopani, from the latter group 38.1% is diagnosed in Vhembe, 35.7% in Mopani and 18.6% in Bohlabela (X^2 ^= 4,538; p < 0.001).

When all the analyses above are limited to those cases definitely originating from Limpopo, all incidence rates are lower, but the differences and trends stay the same. The mean incidence rate was 79.8 per 100,000 person-years (SD 29.1). There was a decreasing trend in the incidence rate over time (X^2 ^for trend = 3,770.9; p < 0.001), from 122.5 in 1998–1999 to 35.7 in 2006–2007. The mean incidence rate in males was higher than in females (89.3 versus 70.6 per 100,000 person-years; X^2 ^= 58.2; p < 0.001). The incidence rate was lowest in 0–4 year olds (50.3), it peaked at the ages of 35–39 years (100.4), and decreased with age from 40 years (to 56.3 for those = 60 years). The incidence rate varied widely between districts; Vhembe (251.2), Mopani (98.0), Bohlabela (80.0), Waterberg (10.6), Sekhukhune (3.9) and Capricorn (3.4)

## Discussion

Over the seasons 1998 – 1999 to 2006 – 2007 the incidence rate of malaria showed a statistically significant decreasing trend in Limpopo Province. This is most likely to a considerable extent attributable to the scaling-up of DDT spraying in the region (in order to combat insecticide-resistant *Anopheles funestus*), with RDT-based rapid case detection contributing, as well as the introduction of ACTs for the treatment of uncomplicated malaria in Limpopo Province in December 2004 [1,21]. Malaria incidence could also be altered due to the regional effect the Lubombo Spatial Development Initiative (LSDI) Malaria Control programme is having. This tri-country initiative has managed to dramatically reduce the incidence of malaria in South Africa (Mpumlanaga and KwaZulu-Natal Provinces), Swaziland and Mozambique (Maputo province). As the LSDI programme has not extended fully to Gaza Province, the province neighbouring Limpopo, the effect on malaria transmission in Limpopo will be limited. As there is also very little movement between the malaria risk areas of Mpumalanga and KwaZulu-Natal, the reduction of malaria cases in these two provinces would have very little effect on Limpopo. It is however envisaged that the extension of the LSDI programme into Gaza Province would have a direct effect on the malaria incidence in Limpopo.

The CFR is fairly stable over the whole period (with a peak in the 2003–2004 season). This might be because the regimen for treating complicated malaria has not been changed. At the time of the study intravenous quinine was the only treatment available in South Africa for patients with severe malaria. The WHO now recommends the more effective treatment with intravenous artesunate for severe malaria in adults in low and moderate transmission areas [22,23]. Furthermore, some say the relatively high CFRs [national target is 0.5% [24]; estimated CFR for the WHO region sub-Saharan Africa is 0.4% [25]] might generally reflect the still thinly distributed health care facilities, and logistical problems, mostly at peripheral clinic level in Limpopo [24]. However, a confidential inquiry conducted in Limpopo a few years ago made clear that PHC at the periphery is actually of a high standard, with excellent management of malaria patients at this level. The main issue was delay in treatment seeking, not because health services were inaccessible, but because of other practices, mainly the use of traditional medicine. Another explanation for the higher observed CFR is that, in contrast to most malaria-endemic areas, malaria in Limpopo is mostly a disease of adults, and the CFRs were high in adult age groups. Among children in Limpopo the CFRs were similar to the CFR of 0.4% mentioned for all of sub-Saharan Africa.

Regarding the distribution of reported malaria cases over the different months of the season; historically the malaria incidence peaks were later in the season in South Africa [9]. The shift to November–January might indicate a change in the dynamics of the malaria epidemiology and transmission patterns in Limpopo.

The incidence rate of malaria in males is higher than in females. The CFR is similar for both sexes. The higher incidence in males might be due to the fact that males are moving around more frequently in the region, mostly for work. This movement might also include movement across the country borders between malaria endemic areas and Limpopo. Migrant workers coming from abroad to Limpopo are also predominantly males. The latter is partly reflected by the fact that the percentage of males is higher in the group of cases that are (probably) not originating from the province.

As can be seen from Table 2 and Figure 3, the malaria incidence rate is related to age. Incidence is lowest in the 0 – 4 year olds and then gradually increases and peaks at the age of 30 – 39 years, after which it gradually decreases again. In populations that lack immunity a predominantly flat age profile is expected [14] which is different from high endemicity malaria areas where the distribution of numbers and severity of malaria episodes is distinctly skewed towards the age group of the under-fives [26]. A study conducted in KwaZulu-Natal, using data from the malaria reporting system for the period mid-1990 to mid-1999, reported on the variation in the pattern of age-specific malaria incidence [12]. In areas of high incidence within the province, incidence rose with age until the late teenage years and either remained constant or decreased afterwards in adults. The authors explained this steadily rising incidence with age during childhood by an increase in chance of infections as children become older. Incidence was lowest in the under five age group possibly because children at this age are indoors at night and benefit from the protection offered by indoor house spraying. As they get older, their sleeping patterns may be less regular and they may therefore be more at risk of infective bites by mosquitoes. Movement further increases, with the higher possibility of getting infected, once individuals start looking for work, which might also include cross border movements and migrants looking for employment in Limpopo. The latter is partly reflected by the fact that the age distribution of the group of cases that are (probably) not originating from the province is somewhat shifted to the higher age categories compared to that of the cases originating from Limpopo.

The CFR increases with increasing age, as malaria tends to be more severe in older people and treatment less successful; a finding very much in line with what is encountered in non-immune elderly travellers returning with malaria [27]. The high CFR in the older age group (which is different from high endemicity malaria areas, where pregnant women and children under five are more at risk of severe and complicated malaria) might also indicate poor treatment seeking behaviour in this group that needs further investigation.

Of the six districts of Limpopo, Vhembe has by far the highest incidence rate, followed by Mopani and Bohlabela. Waterberg, and especially Capricorn and Sekhukune have a much lower incidence rate of malaria. This may be explained by e.g. cross-border movements; climatic factors, which are favourable for the malaria vector, in north-eastern Vhembe and Eastern Mopani, and different from those of the other areas. There are no suspected differences in completeness of reporting between districts.

The CFR in Capricorn and to a lesser extent in Sekhukune seems high compared to that of the other districts (although not statistically significant different). Maybe in areas with a low malaria incidence patients present (too) late to health care facilities due to poor recognition of symptoms and/or the health system does not respond adequately (delayed or inaccurate diagnosis and/or treatment).

As spatial variation of malaria incidence may be considerable, which would have implications for planning, implementing and evaluating malaria control measures [28], more details on local epidemiological patterns down to community level would be helpful.

A limitation of this study is that it is based on routine data from the provincial malaria control programme. This system relies mainly on passive reporting; this means that there is an unknown degree of under-diagnosing and -reporting. The system of passive reporting did not change over the time of the study, so this under-ascertainment should not have affected the trends which were detected. There could be some underreporting due to asymptomatic cases, although their number will be small, as in a population with low-level immunity such as the South African one, most infections lead to clinical symptoms. [11] However, there will at least be some asymptomatic malaria, especially in those districts with high malaria incidence.

Another limitation is that the population estimates do not include migrants from e.g. Mozambique and Zimbabwe, so the denominator of the incidence rates is too small. However, these people are included among the malaria cases. This is partly supported by the fact that the group including cases of whom the location of contracting malaria is unknown, are more most often diagnosed in the three districts (Vhembe, Mopani and Bohlabela) bordering Mozambique and Zimbabwe. As a result the incidence rates might be somewhat overestimated.

## Conclusion

This study gives an overview of the malaria incidence and mortality in Limpopo Province, South Africa, for the seasons 1998–1999 to 2006–2007. In summary, malaria is highly seasonal in Limpopo Province. The mean incidence rate was 124.5 per 100,000 person-years and the mean CFR 1.1% per season. There is a very significantly decreasing trend in the incidence rate over time (p < 0.001). The CFR is fairly stable over the whole period. The mean incidence rate in males is higher than in females (145.8 versus 105.6; p < 0.001); the CFR (1.1%) is similar for both sexes. The incidence rate peaks at the ages of 35–39 years (172.8). The CFR increases with increasing age (to 3.8% for those ≥ 60 years). There is large variation in malaria incidence (up to 70-fold) between the districts in Limpopo Province.

This study provides baseline data about the distribution of malaria in Limpopo Province over time, which will aid future research to refine and evaluate targeted intervention strategies (prevention, diagnosis, treatment). It also answers the need for better epidemiological data over a range of epidemic settings. Malaria control in these areas represents a different challenge from that in endemic settings [14]. This information can guide the development of an individually tailored malaria elimination strategy for Limpopo and other epidemic-prone malaria areas in Africa, by helping to optimize the application of our malaria control tools at hand.

## Competing interests

The authors declare that they have no competing interests.

## Authors' contributions

AAMG designed the study, performed the statistical analysis and drafted the manuscript. PK conceived of the study, participated in the acquisition of data, interpretation of data and revised the manuscript critically. MFSL and MPG both contributed to the analysis and interpretation of data and contributed to the writing of the manuscript. All authors have given final approval of this version to be published.
